# Large Myxoid Ovarian Leiomyoma: A Case Report

**DOI:** 10.1002/ccr3.9658

**Published:** 2024-12-02

**Authors:** Zahra Aminparast, Amirmohammad Khodaei, Zeinab Shakibaee Fard

**Affiliations:** ^1^ Clinical Research Development Center, Imam Reza Hospital Kermanshah University of Medical Sciences Kermanshah Iran; ^2^ Student Research Committee, Medical School Kermanshah University of Medical Sciences Kermanshah Iran

**Keywords:** large ovarian leiomyoma, leiomyoma, myxoid ovarian leiomyoma, ovarian leiomyoma

## Abstract

Ovarian leiomyoma is a rare, benign spindle cell tumor with a favorable prognosis. Myxoid leiomyoma, a rare subtype of leiomyoma, exhibits significant myxoid changes. We present a case study of a rare ovarian myxoid leiomyoma in a young woman. The patient presented with left flank pain and a large abdominal mass upon physical examination. Imaging indicated a solid‐cystic right ovarian mass. The patient underwent a right salpingo‐oophorectomy, and a 5 kg mass with myxoid degeneration was resected. Histopathological and immunohistochemical examination confirmed the diagnosis of ovarian myxoid leiomyoma. We discussed the challenges in the diagnosis of ovarian myxoid leiomyoma and emphasized the importance of accurate diagnosis and appropriate surgical treatment, with a favorable prognosis. This case contributes to the understanding of rare ovarian tumors and highlights the significance of histopathological and immunohistochemical examination in confirming the diagnosis.

**Summary**

- Ovarian leiomyoma is a rare, benign spindle cell tumor with a good prognosis.
- This case emphasizes the coexistence of ovarian leiomyoma and myxoid degeneration, which may present with nonspecific symptoms.
- Accurate differentiation from malignant tumors is crucial and can be achieved through histopathological and immunohistochemical evaluation.

## 1 Introduction

Ovarian leiomyoma is a rare, benign spindle cell tumor that accounts for 0.5%–1.0% of all ovarian tumors and has an excellent prognosis [1].

There is a wide age range, with a mean age of 35 years old, and it is usually observed in women of reproductive age and less commonly in postmenopausal women [1]. It is usually unilateral and rarely bilateral; however, bilateral ovarian leiomyomas have been reported in young women [2, 3].

Most ovarian leiomyomas are found incidentally as ovarian tumors during ultrasound examination or during abdominal surgeries [2, 4]. Clinically, many patients with ovarian leiomyomas may not show any symptoms; however, large ovarian leiomyomas are often symptomatic. They may present with abdominal pain, weight gain, abnormal uterine bleeding, menorrhagia, frequent urination, and vomiting. Some cases may mimic hydronephrosis or acute abdominal pain resembling acute appendicitis [2, 5].

Ovarian leiomyoma is diagnosed by a combination of histopathological evaluation and immunohistochemical studies [2, 3, 4].

Ovarian leiomyoma is similar to uterine leiomyoma and can undergo secondary changes such as hemorrhage, calcification, cystic degeneration, and myxoid degeneration [4, 6].

Myxoid leiomyoma of the ovary is a very rare entity and occurs in 3%–13% of leiomyomas [3]. Myxoid degeneration refers to leiomyomas with myxoid changes constituting more than 50% of the tumoral volume, and it consists of smooth muscle cells with a significant accumulation of hypocellular hyaluronic acid material containing mucin [7].

We report a rare case of ovarian myxoid leiomyoma of large size.

## 2 Case History/Examination

A 36‐year‐old virgin woman with a chief complaint of progressive left flank pain for the past month was referred to the obstetrics and gynecology department. The patient did not have any menstrual bleeding disorder, fever, nausea, vomiting, loss of appetite, dysuria, or urinary frequency symptoms. She did not mention any family history, allergies, or medication history. The patient had no history of pregnancy or any surgical procedures.

The patient's pain had a progressive nature and radiated to the left lower quadrant (LLQ) and left inguinal region. On physical examination, the patient had tenderness in the costovertebral (CV) angle, and a large abdominal mass was found that was firm, large, and bulky, extending from the epigastrium to the lower part of the umbilicus.

It is worth mentioning that the patient's blood pressure was 130/80 mmHg, the heart rate was 90 beats per min, and body temperature was 37°C upon arrival.

## 3 Methods (Differential Diagnosis, Investigations, and Treatment)

Laboratory tests did not show anemia (Hb:14.3 and MCV:88.6), and other laboratory tests including renal function tests, liver function tests, Beta‐hCG titer, ESR, and UA revealed no abnormal findings. Tumor markers were requested for the patient, and CEA and AFP were in the normal range, but CA‐125 was higher than the normal range with titer 151.

**FIGURE 1:**
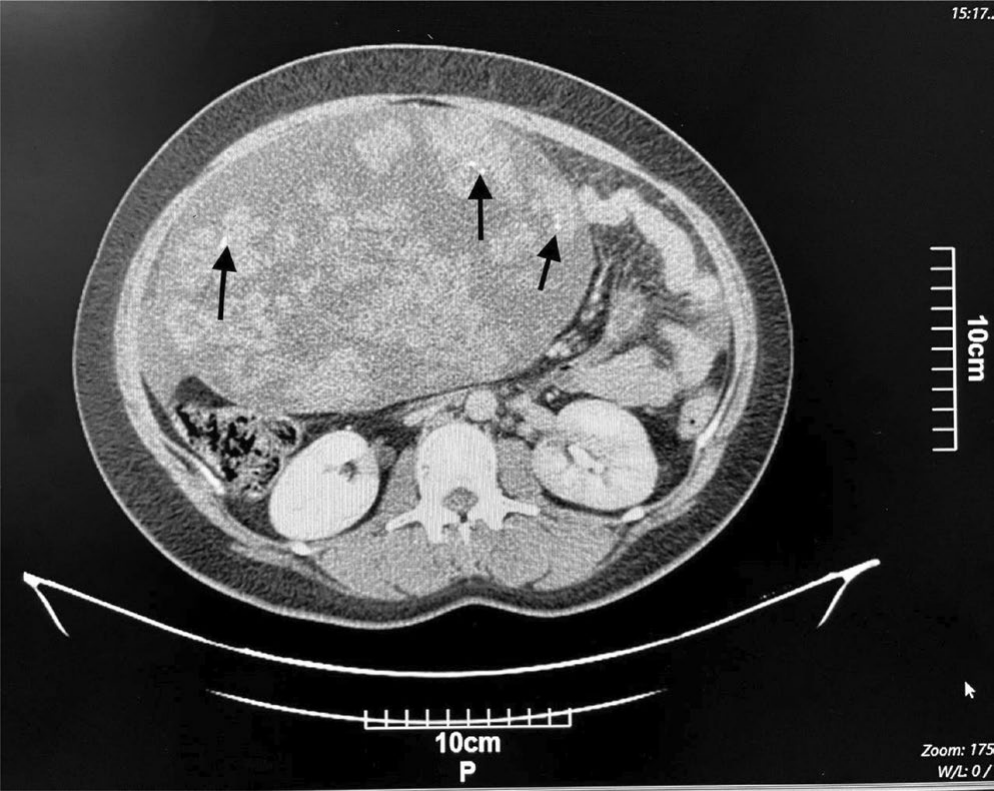
CT scan of the abdomen and pelvis (transverse view) showing solid cystic mass with calcification (black arrows).

**FIGURE 2:**
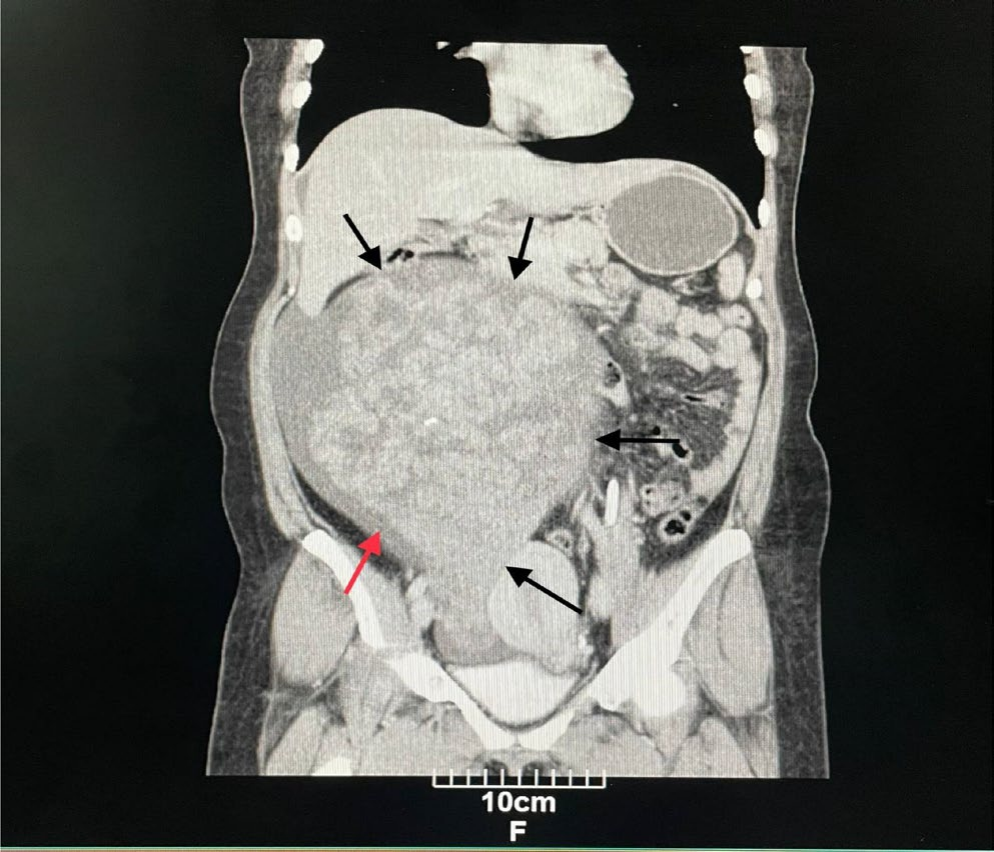
CT scan of the abdomen and pelvis (coronal view) showing huge pelvi‐abdominal mass compressing the intestinal loops and uterus (black arrows) also showing ascites fluid (red arrow).

Computed tomography (CT) of the abdomen and pelvis with IV contrast showed a solid‐cystic huge mass containing several foci of calcification (Figure 1), with the right adnexa origin measuring 260 × 222 × 144 mm with extension into the epigastrium. Based on the findings, a small to moderate amount of ascitic fluid was also observed (Figure 2). The differential diagnosis of malignant ovarian tumor included sex cord stromal tumor, ovarian fibrothecoma, and benign masses including ovarian thecoma and pedunculated subserosal uterine leiomyoma.

The patient underwent right salpingo‐oophorectomy, during which an extended midline incision was made, and after removing the mass, the weight of the mass was reported to be 5 kg. An ovarian mass sample was sent to the pathology department for frozen section diagnosis.

The specimen was received in fresh state and consisted of the solid mass, with the intact surface capsule measuring 30 cm.

**FIGURE 3:**
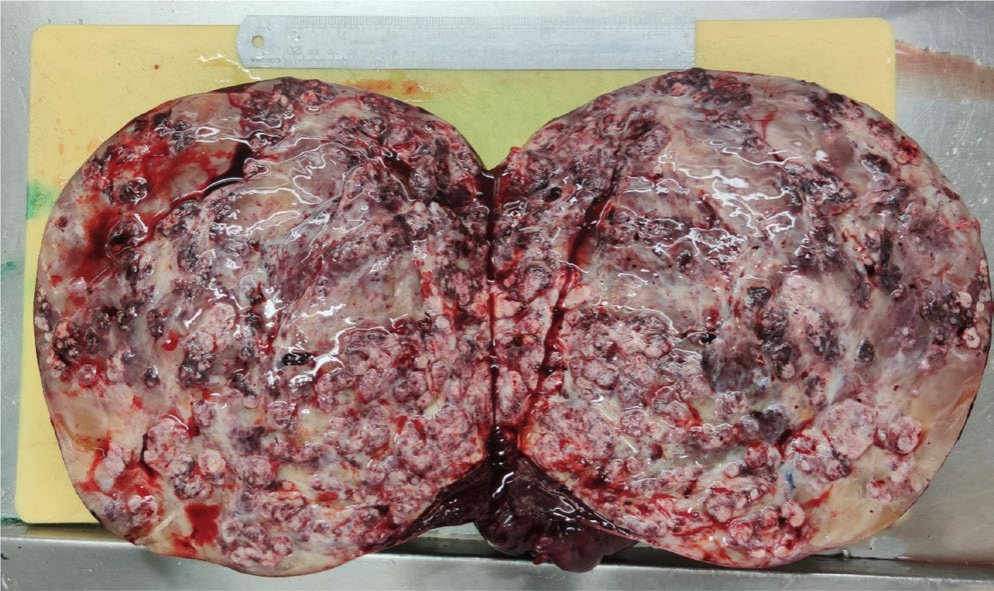
Myxoid leiomyoma, a large mass with several separated cream‐colored whorling areas with hemorrhage and significant myxoid changes.

On cut section, it revealed scattered creamy colored firm area with whorled appearance and abundant myxoid degeneration (more than 50% of tumoral surface) and hemorrhagic areas (Figure 3).

**FIGURE 4:**
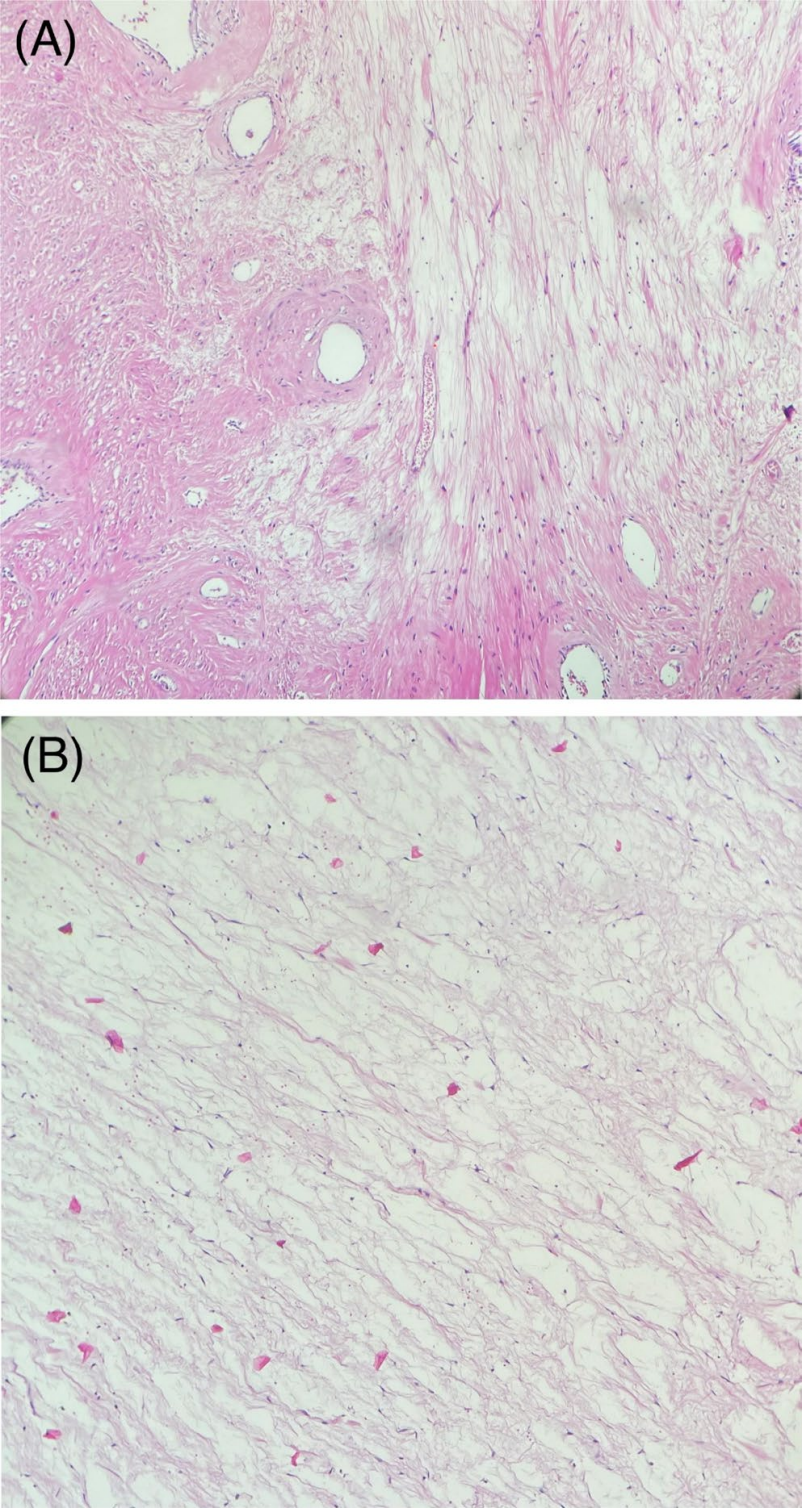
(A) Hematoxylin–eosin stain × 100 photo by author. (B) Hematoxylin–eosin stain × 400 photo by author.

Histologically, frozen sections were diagnosed as spindle cell tumor, and permanent sections revealed areas of intersecting fascicles of uniform spindle cells with cigar‐shaped nuclei and eosinophilic cytoplasm, separated in many areas by myxoid matrix. Also, foci of hemorrhage, calcification, and hyalinization were seen. No evidence of coagulative necrosis, mitosis, or nuclear atypia was observed (Figure 4A,B).

For definitive diagnosis, immunohistochemistry (IHC) including calretinin, SMA, desmin, WT1, vimentin, and Ki‐67 was done, and IHC results were in favor of a benign tumor derived from ovarian smooth muscle.

Based on histomorphology and IHC staining, pathology reported was “ovarian myxoid leiomyoma.”

## 4 Conclusions and Results (Outcome and Follow‐Up)

The patient was discharged from the obstetrics and gynecology department after recovering from salpingo‐oophorectomy with good general condition and stable status. The patient's post‐discharge recommendations included taking cefixime tablets and mefenamic acid capsules.

## 5 Discussion

Leiomyoma is a rare benign ovarian tumor that is often unilateral, with a predilection for the right ovary, especially in symptomatic cases, and most tumors are 5–10 cm in size [6]. However, Agrawal reported the largest ovarian leiomyoma, with about 25 cm diameter [8].

Symptoms of ovarian leiomyoma are usually nonspecific and related to tumor growth and its compressive effects, including lower abdominal distension, frequent urination, constipation, and ascites [2, 4, 5, 6, 8].

Furthermore, ovarian torsion, elevated CA‐125 levels, and Meigs syndrome are associated with an increase in tumor size. Their association with infertility has also been reported [4]. However, these symptoms do not provide enough specific information to distinguish between benign and malignant mesenchymal tumors in a differential diagnosis [9].

Ovarian leiomyomas can be classified into two types: primary ovarian leiomyoma originates from smooth muscle cell in the ovarian hilum vessel wall, and less commonly, it originates from smooth muscle cells in ovarian ligaments, ovarian cortex, and undifferentiated reproductive cells. Secondary ovarian leiomyoma is derived from extra‐ovarian tissue and attaches to the ovary [1, 6].

Estrogen and progesterone play an important role in the growth of leiomyoma, and the size of leiomyoma increases during pregnancy and menstruation [1, 6].

Preoperative diagnosis of ovarian leiomyoma with imaging methods is sometimes difficult, especially in tumors with cystic, hemorrhagic, and calcified degeneration, which may be misdiagnosed as malignant tumors [2]. In these cases, magnetic resonance imaging (MRI) is the main diagnostic tool and can show details of anatomical features [1, 6].

CT imaging is also used in diagnosing ovarian tumors when a more detailed and precise imaging of the abdomen and pelvis is needed beyond what sonography can provide. CT imaging can provide a clearer view of the size, location, and characteristics of the tumor, as well as any potential spread to surrounding tissues or organs. It can also be useful in cases where the patient is unable to undergo a transvaginal ultrasound due to medical conditions or patient preference. Although CT imaging primarily provides anatomical information, it may not always show the functional characteristics of ovarian tumors, such as blood flow or metabolic activity [1, 2, 3, 4, 5, 6].

Macroscopically, tumors are often solid, firm, and round with a smooth surface; on cut section, they have a white or gray‐tan whorled appearance with secondary changes such as hemorrhage, calcification, and myxoid degeneration, especially in the large tumors [6, 8, 10]. The majority of the reported tumors were able to move easily, although in some cases, they may become attached to the omentum, intestine, or uterus [8].

Microscopically, conventional leiomyoma consists of irregular bundles of spindle‐shaped cells with elongated blunt nuclei or cigar‐shaped nuclei, without coagulative necrosis, nuclear atypia, or increased mitotic activity. A correct diagnosis of ovarian leiomyoma requires recognizing the smooth muscle nature of the tumor [8].

The pathology report of the female genital tract leiomyoma is similar to that of the uterine leiomyoma. Myxoid leiomyoma was first reported in female genital tract neoplasia by Tavassoli and Norris in 1979 in a vulvar case [11].

Myxoid smooth muscle lesions, based on infiltrative border, atypia, tumor cell necrosis, and mitoses, are classified into: myxoid leiomyoma, the myxoid smooth muscle tumor with uncertain malignant potential, and myxoid leiomyosarcoma. According to the 5th edition of the female genital tract classification by WHO, myxoid leiomyoma is a well‐circumscribed tumor that shows spindle‐shaped smooth muscle cells with eosinophilic cytoplasm and cigar‐shaped nuclei in the myxoid acid mucin stroma; the cells lack cytological atypia and mitotic activity [12].

Differential diagnosis of ovarian myxoid leiomyoma includes sclerosing stromal tumor and ovarian myxoma, and the correct diagnosis requires the confirmation of the smooth muscle nature of the tumor cell by immunohistochemistry(IHC). IHC shows positive results of vimentin, desmin, and SMA and negative results of inhibin [6].

Rarely, leiomyomas may undergo myxoid degeneration, adding complexity to the diagnostic process and making it difficult to differentiate between conditions like choriocarcinoma, sarcomatous transformation, or even hydatid cysts. Therefore, accurate diagnosis is essential to avoid misdiagnosing them as malignant tumors [10].

The follow‐up of patients with small tumors is recommended, but radical surgical treatment has been suggested for patients with a tumor size of 3–4 cm to prevent ovarian destruction by the tumor. However, bilateral salpingo‐oophorectomy and hysterectomy may be considered in middle‐aged and older adult patients [2].

## 6 Conclusions

Ovarian leiomyoma is a rare ovarian tumor in which myxoid degeneration is one of its uncommon changes. Clinical diagnosis is difficult and is often misdiagnosed as malignant ovarian tumors. When a solid mass is detected in the adnexal tissues, ovarian leiomyoma should be considered. The diagnosis of this tumor through preoperative imaging is challenging. In such cases, a final diagnosis is only made after histopathological and immunohistochemical studies. Unilateral salpingo‐oophorectomy or unilateral oophorectomy is the preferred treatment for patients of all ages with ovarian leiomyoma and is associated with a good prognosis.

By presenting this case, we aim to emphasize a rare presentation of ovarian leiomyoma and underscore the significance of histopathological and immunohistochemical evaluations to avoid misdiagnosis in the future.

## Author Contributions

**Zahra Aminparast:** investigation, resources, supervision, writing – original draft. **Amirmohammad Khodaei:** investigation, project administration, software, writing – review and editing. **Zeinab Shakibaee Fard:** investigation, software, supervision, writing – review and editing.

## Ethics Statement

This manuscript does not include personal or medical details about any identifiable individual. The patient's brother provided consent for the writing and publication of this article.

## Consent

Apart from age and gender, no identifying details were included in the manuscript. The patient's brother provided written consent for the utilization of their hospital records and imaging as per the journal's patient consent guidelines.

## Conflicts of Interest

The authors declare no conflicts of interest.

## Data Availability

The data that support the findings of this study are available on request from the corresponding author. The data are not publicly available due to privacy or ethical restrictions.
